# Progress of MRI Radiomics in Hepatocellular Carcinoma

**DOI:** 10.3389/fonc.2021.698373

**Published:** 2021-09-20

**Authors:** Xue-Qin Gong, Yun-Yun Tao, Yao–Kun Wu, Ning Liu, Xi Yu, Ran Wang, Jing Zheng, Nian Liu, Xiao-Hua Huang, Jing-Dong Li, Gang Yang, Xiao-Qin Wei, Lin Yang, Xiao-Ming Zhang

**Affiliations:** ^1^Medical Imaging Key Laboratory of Sichuan Province, Department of Radiology, Medical Research Center, Affiliated Hospital of North Sichuan Medical College, Nanchong, China; ^2^Department of Hepatocellular Surgery, Institute of Hepato-Biliary-Intestinal Disease, Affiliated Hospital of North Sichuan Medical College, Nanchong, China; ^3^School of Medical Imaging, North Sichuan Medical College, Nanchong, China

**Keywords:** hepatocellular carcinoma, magnetic resonance imaging, intravoxel incoherent motion, radiomics, immune checkpoint inhibitors, target therapies, therapeutic response, diagnosis

## Abstract

**Background:**

Hepatocellular carcinoma (HCC) is the sixth most common cancer in the world and the third leading cause of cancer-related death. Although the diagnostic scheme of HCC is currently undergoing refinement, the prognosis of HCC is still not satisfactory. In addition to certain factors, such as tumor size and number and vascular invasion displayed on traditional imaging, some histopathological features and gene expression parameters are also important for the prognosis of HCC patients. However, most parameters are based on postoperative pathological examinations, which cannot help with preoperative decision-making. As a new field, radiomics extracts high-throughput imaging data from different types of images to build models and predict clinical outcomes noninvasively before surgery, rendering it a powerful aid for making personalized treatment decisions preoperatively.

**Objective:**

This study reviewed the workflow of radiomics and the research progress on magnetic resonance imaging (MRI) radiomics in the diagnosis and treatment of HCC.

**Methods:**

A literature review was conducted by searching PubMed for search of relevant peer-reviewed articles published from May 2017 to June 2021.The search keywords included HCC, MRI, radiomics, deep learning, artificial intelligence, machine learning, neural network, texture analysis, diagnosis, histopathology, microvascular invasion, surgical resection, radiofrequency, recurrence, relapse, transarterial chemoembolization, targeted therapy, immunotherapy, therapeutic response, and prognosis.

**Results:**

Radiomics features on MRI can be used as biomarkers to determine the differential diagnosis, histological grade, microvascular invasion status, gene expression status, local and systemic therapeutic responses, and prognosis of HCC patients.

**Conclusion:**

Radiomics is a promising new imaging method. MRI radiomics has high application value in the diagnosis and treatment of HCC.

## Introduction

Hepatocellular carcinoma (HCC) is the sixth most common cancer and the third leading cause of cancer-related death worldwide (1). Although the diagnostic criteria of HCC continue to improve, its prognosis remains unsatisfactory (2). In addition to certain factors, such as tumor size and number and vascular invasion displayed on traditional imaging, some histo-pathological features and gene expression parameters are also important in the prognoses of patients with HCC. However, many current staging systems for HCC have not taken into consideration the above-mentioned histopathological features or genetic traits beyond the size and number and vascular invasion of the tumor (3, 4). Most parameters are based on postoperative pathological examinations, which cannot help with preoperative decision-making. To better stratify HCC patients before surgery, make more accurate treatment decisions, and improve the prognoses of patients, there is an urgent need for a noninvasive method that can accurately predict the histo-pathological features and gene expression parameters before surgery. The rapid development of artificial intelligence has played an important role in personalized precision medicine (5). Radiomics, a new technology, can transform the potential histopathological and physiological information in images into high-dimensional quantitative image features that can be mined (6, 7).The study of radiomics will contribute to the early diagnosis and treatment of HCC and ultimately improve survival (8, 9). In recent years, many studies have confirmed the application values of magnetic resonance imaging (MRI) radiomics in the diagnosis and differentiation (10, 11), histological grading (12, 13), microvascular invasion (MVI)assessment (14, 15), radiogenomics (16, 17),prediction of relapse and prognosis after surgical resection (18–20), response to transarterial chemoembolization(TACE) (21, 22) and systemic treatment efficacy of HCC (23).

To better understand the research hotspots and trends of MRI radiomics in HCC, we used PubMed to identify important recent publications on MRI radiomics in HCC, selected research articles and reviews and used bibliometric method to visually analyze the countries, institution, authors, and keywords of MRI radiomics in HCC. Meanwhile, this study reviews the radiomics workflow from image acquisition and reconstruction, segmentation, feature extraction, feature selection and modeling to model validation, and the research progress of MRI radiomics in HCC.

## Bibliometrics of MRI Radiomics in HCC

**Figure 1:**
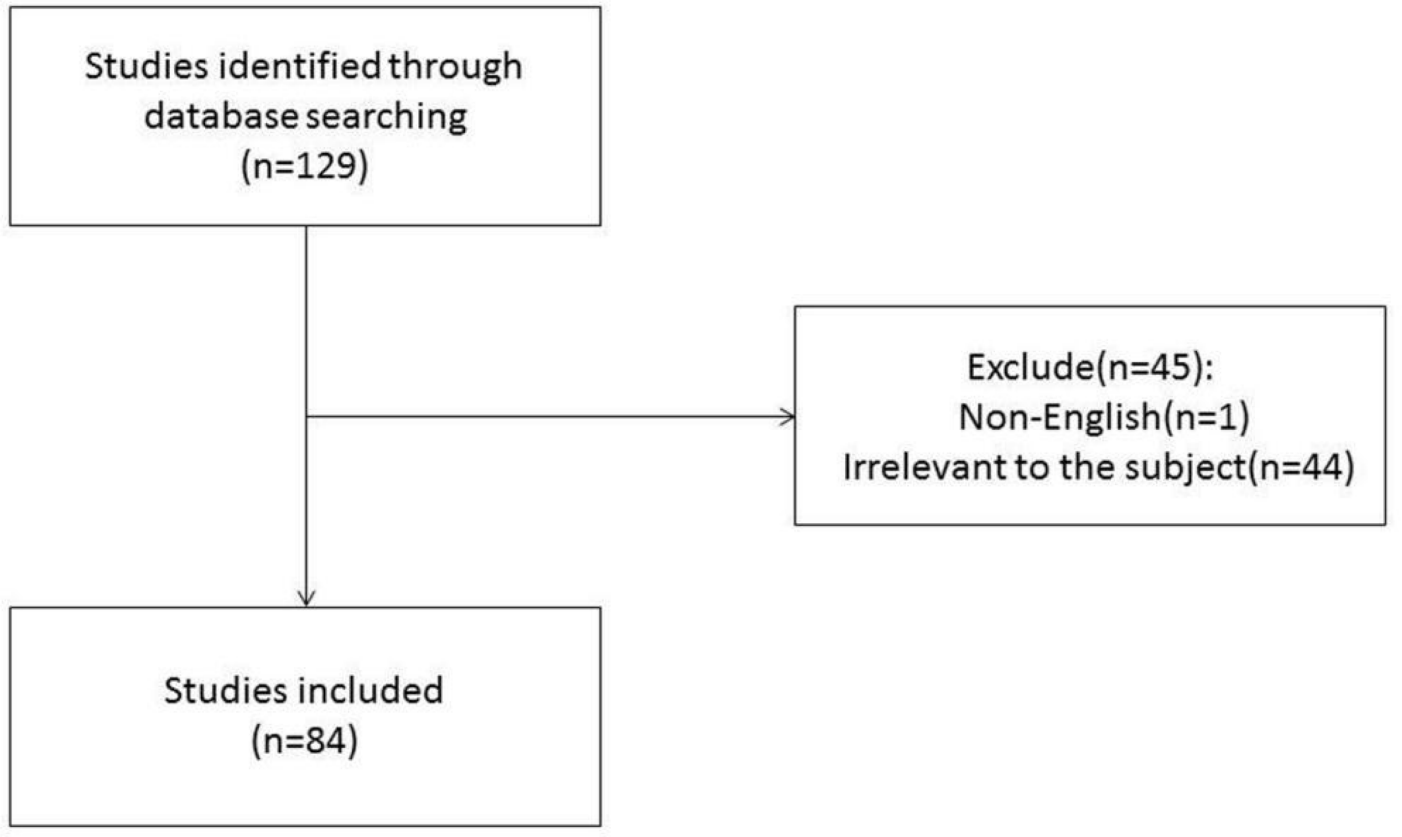
Flow diagram of study selection.

**Figure 2:**
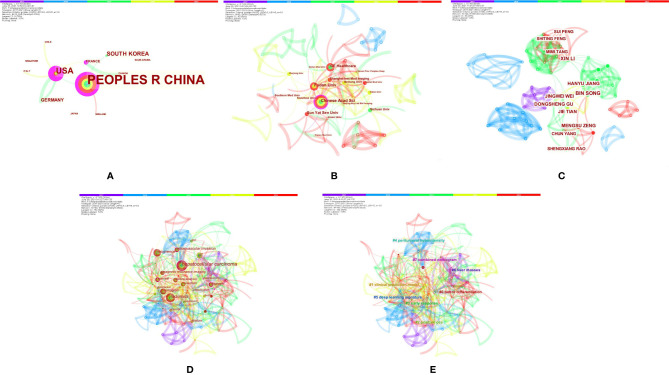
Bibliometrics of magnetic resonance imaging radiomics in hepatocellular carcinoma. **(A)** Co-occurrence map of countries. **(B)** Co-occurrence map of institutions. **(C)** Co-occurrence map of authors. **(D)** Terms in theco-occurrence network. **(E)** Terms in theco-occurrence clusters.

The authors conducted a literature review using PubMed to identify important recent publications and determine the current status of radiomics in HCC. A comprehensive list of MeSH terms and keywords was included in the search: HCC, MRI, radiomics, deep learning(DL), artificial intelligence, machine learning, neural network, texture analysis, diagnosis, histopathology, microvascular invasion, surgical resection, radiofrequency, recurrence, relapse, TACE, targeted therapy, immunotherapy, therapeutic response, and prognosis. The inclusion criteria were as follows: (1) original research articles and review articles published in the English language between May 2017 and June 2021; (2) literature related to MRI radiomics or DL; and (3) literature related to diagnosis and differentiation, histological grading, MVI assessment, radiogenomics, prediction of relapse and prognosis after surgical resection, response to TACE and systemic treatment efficacy of HCC. Articles that were not published in English and those containing irrelevant information on the subject were excluded. We also excluded articles that were published before May 2017 and after June 2021. In total, 129 articles were ultimately retrieved. After screening the titles, abstracts and full texts (if appropriate), only 84 papers met the criteria for inclusion (**Figure 1**). Those 84 papers were then downloaded with the record content of “Full Record and Cited References” and the file format of “Plain Text”. As CiteSpace can only recognize files named “download *.txt”, the files were renamed accordingly. The bibliometric software CiteSpace5.7.R2 (64 bits) was utilized for this study to visually analyze the countries, institution, authors, and keywords draw relevant charts. The articles originated from a total of 12 countries, and the top five countries were China (24), the USA (13), South Korea (5), Germany (3), and France (2). A total of 30 institutions published manuscripts independently or cooperatively. The top five institutions were the Chinese Academy of Sciences (12), Fudan University (10), GE Healthcare (8), Sun Yat-Sen University (7), and Sichuan University (5). Bin Song and Xin Li were the most prolific authors. Meng-Su Zeng, Jie Tian, and Dong-Sheng Gu were also active in this field. “Hepatocellular carcinoma” was the most important term, followed by “radiomics”, “recurrence”, and “microvascular invasion”. According to the link strength of keyword cooccurrence, the network was divided into eight clusters, and the largest cluster was “tumor differentiation (#0)” (**Figure 2**).

## Radiomics Workflow

Radiomics extracts high-throughput features from images and transforms imaging data into high-resolution mining data spaces through machine learning (25). Quantitative radiological data can therefore be extracted and applied to clinical decision-making (25). The workflow of radiomics usually includes five steps (6), which are described below.

## Image Acquisition and Reconstruction

Imaging techniques that can be used for radiomics include MRI, computed tomography (CT), positron-emission tomography, and ultrasound. Among them, MRI has the advantage of depicting more soft-tissue features. Radiomics is an imaging analysis method; thus, it is vital to standardize high-quality images (26–28). This makes it necessary to preprocess the imaging data; otherwise, a widely promoted standard scanning protocol is needed to reduce the variability in radiomic features and improve the performance of radiomic models (25, 29).

## Image Segmentation

Manual, automatic, and semiautomatic segmentation are often used to segment the volume or region of interest in a target tissue (30). Manual segmentation is most reliable, but it involves intraobserver and interobserver variability. Its labor and time cost are high. The segmentation of an image often requires multiple clinicians or the same clinician at multiple times. The intraobserver and interobserver variability can be improved by screening the intraobserver and interobserver consistency. The purpose of automatic segmentation is to mark the regions of interest automatically by a computer. Semiautomatic segmentation involves manual corrections. Automatic segmentation algorithms include image segmentation based on thresholds, image segmentation based on region growing, and image segmentation based on edge detection. Some classical algorithms perform well at delineating liver lesions (31, 32).

## Image Feature Extraction

Image features include semantic features and nonsemanticfeatures (33). Semantic features include qualitative (shapes, boundaries, etc.) and quantitative features, and their analysis depends on the radiologist’s knowledge. Nonsemantic features are quantitative descriptors extracted from tissues of interest, including shape and statistical features (34). The shape features of objects in images include topological features, distances, perimeters, areas, geometric features, and descriptions of shape and orientation. Statistical features can be further divided into first-order, second-order, and high-order features. First-order features are usually called density features, which involve gray-level histogram information simply describing the global distribution of gray levels in an image. Such features cannot describe the local distribution of gray levels in an image or the spatial position of each gray level (35). Second-order features are often called texture features. These reflect the relationships between adjacent voxels. High-order features are usually called filtering features and are generated by wavelet and Laplacian Gaussian filtering, for example, in addition to first-order and second-order features.

## Feature Selection and Modeling

Many features can be extracted from a high-throughput image, but using all the features to analyze an image will lead to overfitting. The best features can be selected by dimensionality reduction to improve the efficiency of the model. The methods of feature selection can be divided into three categories: filter, wrapper, and embedded (36). The goal of radiomics is to establish a prediction model for clinical outcomes from selected features. The modeling methods include logistic regression, k-nearest neighbor, decision trees, ensemble learning, and support vector machines. It is recommended to test the effectiveness of several forecasting models to select the model with the best performance (37).

## Model Validation

The prediction model can be validated by internal cross-validation, such that the model can be further optimized and the prediction performance can be maximized. Validation of the model should be carried out in a separate cohort (37). For differentiation analysis, the receiver operating characteristic (ROC) curve is the most commonly used method to evaluate the performance of the model. The area under the ROC curve (AUC) or the sensitivity and specificity of the model can be used to evaluate whether the model can predict clinical outcomes. For survival analysis, the concordance index (C-index) and the time-related ROC curve are usually used for validation (38).

## Diagnosis and Differentiation

At present, the diagnosis of HCC is mainly based on imaging methods such as MRI, CT, and ultrasound. Because HCC has a typical enhancement mode, contrast-enhanced CT and dynamic contrast-enhanced MRI play important roles in the diagnosis of HCC (39–42). The European Association for the Study of the Liver standard (40) and the Liver Imaging Reporting and Data System (43) are widely recognized. However, the evaluation of imaging features may be subjective because radiologists have different experiences and different familiarities with the system (44, 45). Radiomics has important application value in the diagnosis of solid tumors because it uses advanced image processing technology to extract high-throughput data and quantitative analysis of tumor behavior and heterogeneity (6, 46–51).

Radiomics signatures based on conventional precontrast T1-weighted imaging, postcontrast T1-weighted imaging, T2-weighted imaging, diffusion-weighted imaging (DWI), and intravoxel incoherent motion (IVIM), whether alone or in combination with clinical data, are all valuable for HCC differentiation (52–59), and their differentiation efficiency is almost equal to that of experienced radiologists (10-year experience) (52). HCC, intrahepatic cholangiocarcinoma (ICC), and HCC-ICC have common risk factors (60, 61), and their typical qualitative MRI features may overlap (24, 62–64). Therefore, the conventional MRI diagnosis of HCC is still uncertain. According to Liu et al. (54), the imaging features extracted from MR images have great potential to differentiate combined hepatocellular cholangiocarcinoma from cholangiocarcinoma and HCC, showing a maximum AUC of 0.77. Recently, Zhu et al. (56) studied the application value of histogram features on IVIM-DWI in the differential diagnosis of HCC. They found that the histogram parameters of IVIM-DWI could distinguish hepatic hemangiomas, hepatic cysts, and HCC and that the volume of the pseudodiffusion coefficient and perfusion fraction had better diagnostic value than other histogram parameters (56).

In recent years, DL technology has been developed and has achieved excellent performance in the classification of hepatic lesions (65–71). Hamm CA et al. (65) developed a proof-of-concept convolutional neural network (CNN)-based DL system and classified 494 hepatic lesions from six categories on MRI. The system demonstrated 92% accuracy, 92% sensitivity and 98% specificity, and their results showed a 90% sensitivity for classifying HCC compared to 60%/70% for radiologists.

## Histological Grading

The histological grading of HCC is key to determining the best treatment scheme and prognosis of a patient. High-grade HCC patients have a higher intrahepatic relapse rate than low-grade HCC patients (72, 73), and most high-grade HCC patients need larger safe resection margins and more frequent postoperative follow-up visits (74, 75). The radiomic features of precontrast T1-weighted imaging, postcontrast T1-weighted imaging, and T2-weighted imaging, whether alone or in combination with clinical data (76), are all valuable for identifying poorly differentiated HCC (13, 76–80). In addition, recent studies have shown the application value of functional MRI radiomics based on IVIM-DWI in predicting the pathological grade of HCC (12, 81, 82). Shi et al. (82)performed MRI on 52 HCC patients and extracted histogram indices from IVIM parameter maps. Eighteen IVIM histogram indices showed the capacity to differentiate histopathological grades. By establishing a diagnostic model based on logistic regression and integrating different histogram indices showing significant differences between different subgroups, the maximum diagnostic power for distinguishing HCC histological grades was obtained (AUC=0.917). This study indicated that histogram indices extracted from IVIM parameter maps had great potential in predicting histopathological grade (82). Geng et al. (12) extracted 107 radiomic features from SWI images of 53 HCC patients and Spearman correlation coefficients were used to evaluate the correlation between SWI radiomic features and histopathology. They found that the SWI radiomic features were significantly correlated with histopathological grades.

## MVI

MVI is diagnosed depending on postoperative tissue specimens, but detection by conventional imaging is difficult. The presence of MVI indicates that the tumor has strong biological invasiveness, which can increase the relapse rate of HCC more than fourfold (83, 84). Accurate preoperative prediction of MVI of HCC can help doctors adjust treatment strategies in a timely manner (such as expanding the resection range), optimize treatment plans, reduce the risks of postoperative relapse, and improve the prognosis (84, 85). Enhanced MRI is helpful to predict MVI in HCC (83, 86–91). MRI-based radiomics (15, 92–102) and DL systems (103–106) have shown good performance in predicting MVI in HCC. The increase in clinicopathological risk factors and qualitative imaging features can improve the prediction efficiency of the model (14, 98, 107, 108). Li et al. (101) found that tumor volume–based IVIM histogram analysis can be used to predict MVI and that the fifth percentile of the true diffusion coefficient is most beneficial to predict MVI of HCC. Zhang et al. (107) extracted imaging features based on preoperative multimodal MR images and constructed an MVI prediction model (combined model) by combining the clinical features and qualitative imaging features of patients with HCC. The AUC in the validation cohort of their combined model was 0.858, which was higher than the AUC (0.820) in the validation cohort of the model constructed from individual radiomic features, indicating that the prediction efficiency of the combined model was higher. Song D et al. (104) predicted MVI using radiomics and DL in 601 patients with HCC based on preoperative MRI. Their results showed that the radiomics model achieved an AUC of 0.731, the DL model based only on MRI images achieved an AUC of 0.915, and a DL model combined with clinical parameters achieved an AUC of 0.931. These studies indicated that the model combining radiomics, DL, and clinical parameters showed the best predictive performance.

## Radiogenomics

The biological behavior of a tumor is closely related to its gene expression profile. Biopsy is a widely used method to evaluate gene expression before surgery, but biopsy is an invasive examination that may cause bleeding and other complications. Therefore, patients are often unwilling to undergo this examination. In recent years, radiogenomics has gradually become more widely applied in HCC research. The purpose of radiogenomics is to determine the relationship between semantic and quantitative image data and genomic and molecular measurements, thus constructing correlation diagrams related to results or other clinical measurements (33, 109, 110). Segal et al. (111) evaluated the correlation between radiogenomic features and the liver cancer gene phenotype and reported that 78% of liver cancer gene expression profiles could be reconstructed by this combination of features. To date, radiogenomics studies have described the semantic features obtained from MRI (112–115). MRI radiogenomics has the value of predicting gene features with prognostic and therapeutic significance (16, 17, 116–123). Taouli et al. (113) found that there was a strong connection between imaging features, such as the “infiltrative pattern”, “mosaic appearance”, and “presence of macrovascular invasion”, and an aggressive genomic signature determined previously. Shi et al. (82) found that histogram indices extracted from IVIM parameter maps could predict Ki-67 expression. Jun et al. (124) used an immunohistochemical method to detect the expression of programmed cell death-1 (PD-1) and programmed cell death ligand-1 (PD-L1) in 98 ICC patients and extracted radiological features from the arterial phase and portal venous phase of preoperative MR images. The results indicated that the AUCs of the models for predicting PD-1 and PD-L1 expression were 0.897 and 0.897, respectively. The prognoses of PD-1-positive and PD-L1-positive patients were worse than those of PD-1-negative and PD-L1-negative patients, and their 5-year survival rates were 12.5%, 48.3%, 21.9%, and 39.4%, respectively (P < 0.05). The results indicated that MRI radiomics could be used as a noninvasive biomarker to evaluate the expression of PD-1 and PD-L1 and the prognosis of ICC patients (**Table 1**).

**Table 1:** Summary of radiogenomics studies.

Ref.	Year	Country	Subject number	Key findings
Shi G et al. (82)	2020	China	52	IVIM histogram metrics can predict expression of the cell proliferation marker Ki-67.
Hectors SJ et al. (116)	2020	United States	48	Radiomics features extracted from MR images correlate with quantitative expression of the immune markers CD3, CD68 and CD31and expression of the immunotherapy targets PD-L1 at the protein level, as well as PD1 and CTLA4 at the mRNA level.
Wang W et al. (17)	2020	China	227	The radiomics-based model performs better than the clinico-radiological model for predicting biliary-specific marker CK19 status of HCC.
Gu D et al. (117)	2020	China	293	The MRI-based radiomics signature is significantly related to GPC3positivity (a prognosis factor, was associated with metastasis and recurrence after resection) in patients with HCC.
Ye Z et al. (118)	2019	China	89	Texture analysis on preoperative enhanced MRI can be used to predict the status of the cell proliferation marker Ki-67 after curative resection in patients with HCC.
Fan Y et al. (16)	2021	China	133	Texture analysis based on enhanced MRI can help identify VETC-positive HCC (histological vascular pattern, micrometastases, early recurrence and poor prognosis).
Li Y et al. (119)	2019	China	83	Texture analysis of multiphase MRI images is helpful for predicting expression of the cell proliferation marker Ki-67 in HCC.
Wang HQ et al. (120)	2019	China	86	Texture analysis based on MRI can help identifyCK19-positive HCC(tends to be related to a worse prognosis).
Fan Y et al. (121)	2021	China	151	A combined model including artery phase radiomics score and serum AFP levels based on enhanced MRI can preoperatively predict expression of the cell proliferation marker Ki-67 in HCC.
Huang X et al. (122)	2019	China	100	MRI radiomics features can be used to preoperatively differentiate dual-phenotype HCC from CK7- and CK19 (markers of cholangiocellular carcinoma) -negative HCC.
Chen S et al. (123)	2019	China	207	Radiomics obtained from enhanced MRI can help predict the immunoscore (density of CD3+ and CD8+ T cells) in HCC.

## Prediction of Relapse and Prognosis After Surgical Resection

Surgical resection is still the main treatment for patients with early HCC (125). However, tumor relapse is still the main cause of postoperative death, and the 5-year relapse rate after surgery is close to 70% (126). Improving the ability to preoperatively identify these high-risk patients will guide surgical management, postoperative monitoring, and treatment intervention (127, 128). The radiomic model based on preoperative MRI can be used as a new tool to predict early relapse (18, 19, 129–134), relapse-free survival (135) and overall survival (OS) (136, 137) in patients with HCC after surgery. Hui et al. (130) used preoperative MRI to extract 290 texture parameters to predict the relapse of HCC patients within 730 days after surgical resection. The results showed that the prediction accuracy of texture features based on dynamic contrast-enhanced MRI in the equilibrium phase was 84%. Combining clinical, laboratory, and radiomic data can improve the performance of quantitative models (20, 129, 135, 136, 138). According to Kim et al. (135), the combined clinical and radiomic model had the same performance as the clinicopathological model in predicting early relapse. Zhang et al. evaluated the effectiveness of contrast-enhanced MRI radiomic features in predicting the OS of HCC patients after resection. Their results showed that preoperative clinical features and semantic imaging features were significantly correlated with survival rate; the Barcelona Clinic Liver Cancer stage, uneven tumor margin, and combined rad-score were independently correlated with OS; and the combined model incorporating radiological and radiomic features had a better prediction performance than the clinic-radiological model (136).

## Prediction of Response to TACE

TACE is recognized as an effective treatment for advanced HCC (125), but its long-term efficacy needs to be further improved (139–141). MRI radiomics can be used to predict the response to TACE treatment and provide a reference for the formulation of individualized treatment plans (21, 22, 142–148). Sun et al. (142)predicted the risk of early postoperative progression based on multiparameter MRI data before TACE. The results showed that the AUC of the model based on DWI features was 0.786 and 0.729 when b=0 and b=500, respectively, followed by the AUC of T2-weighted imaging features (0.729) and the apparent diffusion coefficient (0.714). Compared with any single MRI signal, the MP-MRI signal had a higher AUC, at 0.800. Song et al. (143) revealed that their combined model incorporating radiomic features and clinical radiation risk factors had the best predictive value (C = 0.802).

## Prediction of the Systemic Treatment Efficacy

The treatment of HCC has been a challenge. Systemic therapies for HCC are current research hotspots. Targeted therapy with sorafenib (149) and lenvatinib (150) and immunotherapy with immune checkpoint inhibitors, especially antibodies against PD-1/PD-L1 pathway members (nivolumab and pembrolizumab), have achieved excellent clinical results (151–158). These results strongly indicated that immune checkpoint inhibitor-based strategies will soon be primary method in the treatment of advanced HCC, and immunotherapy will introduce a new era of HCC therapy. Traditional contrast-enhanced CT and MRI, including functional imaging, are the most commonly used biomarkers for evaluating the therapeutic response in clinical practice (159–172). Research based on contrast-enhanced CT and MR images has shown the value of radiomics and DL in predicting systemic treatment efficacy for advanced HCC (23, 173–175). Mulé et al. (174) analyzed the CT texture features of 92 patients before receiving sorafenib and found that the entropy of portal phase-derived entropy at fine texture scales was an independent predictor of OS, which was confirmed in their validation cohort. Yuan et al. (173) established a radiomics nomogram and measured its ability to evaluate the therapeutic efficacy of anti-PD-1antibodies in the treatment of HCC by combining pretreatment contrast-enhanced CT images and clinical risk factors. The results indicated that the AUCs of the radiomics nomogram were 0.894 and 0.883 in the training and validation cohorts, respectively.

In recent years, MRI radiomics has gradually become more widely applied to systemic treatment evaluation of brain tumors (176, 177). There are still no reports on using MRI radiomics to evaluate the systemic treatments of patients with HCC. We believe that as research progresses, MRI radiomics will play an important role in the evaluation of systemic treatments for HCC in the near future.

## Conclusion

As a new technology, radiomics can improve the diagnosis and differentiation of HCC, as well as predictions of the stage, histological grade, MVI, gene expression, treatment response, and prognosis of HCC. This is because it allows us to analyze the relationship between high-dimensional quantitative imaging features and clinical and genetic data. Moreover, it is a powerful tool for making personalized treatment decisions before surgery. With the rapid development of targeted therapy and immunotherapy for HCC, radiomics is expected to become a reliable radiological marker for predicting the therapeutic targets and therapeutic responses of HCC patients.

There are still some challenges and limitations in the clinical application of radiomics. First, a key challenge is to ensure that the academic community can obtain high-quality radiological and clinical resources that involve the establishment and promotion of imaging and clinical data acquisition protocols. Second, the analytical methods of radiomics need to be standardized. Third, many radiomics studies are retrospective, whereas a prospective research design is ideal. As technology advances and research progresses, MRI radiomics will play a more important and even irreplaceable role in the diagnosis and treatment of HCC.

## Author Contributions

X-QG and LY wrote the paper. Y-YT, Y-KW, NinL, XY, RW, JZ, and GY contributed to the literature search and manuscript preparation. NiaL and X-HH revised the paper. X-QW performed the data analysis and created graphs. X-MZ and J-DL designed the research. All authors contributed to the article and approved the submitted version.

## Funding

This work was supported by the Project of Medical Association of Sichuan Province (No. S20070) and the Project of City-University Science and Technology Strategic Cooperation of Nanchong City (North Sichuan Medical College) (No. 20SXQT0324).

## Conflict of Interest

The authors declare that the research was conducted in the absence of any commercial or financial relationships that could be construed as a potential conflict of interest.

## Publisher’s Note

All claims expressed in this article are solely those of the authors and do not necessarily represent those of their affiliated organizations, or those of the publisher, the editors and the reviewers. Any product that may be evaluated in this article, or claim that may be made by its manufacturer, is not guaranteed or endorsed by the publisher.
